# Biomedical Applications for Gold Nanoclusters: Recent Developments and Future Perspectives

**DOI:** 10.1186/s11671-018-2725-9

**Published:** 2018-09-26

**Authors:** Navdeep Kaur, Robby Nur Aditya, Arshdeep Singh, Tsung-Rong Kuo

**Affiliations:** 1 School of Pharmacy, College of Pharmacy, Taipei Medical University, Taipei, 11031 Taiwan; 2 International Ph.D. Program in Biomedical Engineering, College of Biomedical Engineering, Taipei Medical University, Taipei, 11031 Taiwan; 3 Graduate Institute of Nanomedicine and Medical Engineering, College of Biomedical Engineering, Taipei Medical University, Taipei, 11031 Taiwan

**Keywords:** Gold nanoclusters, Imaging, Detection, Therapy; ligand, Small molecules, Polymers, Biomacromolecules

## Abstract

Gold nanoclusters (AuNCs) have been extensively applied as a fluorescent probe for biomedical applications in imaging, detection, and therapy due to their unique chemical and physical properties. Fluorescent probes of AuNCs have exhibited high compatibility, superior photostablility, and excellent water solubility which resulted in remarkable biomedical applications for long-term imaging, high-sensitivity detection, and target-specific treatment. Recently, great efforts have been made in the developments of AuNCs as the fluorescent probes for various biomedical applications. In this review, we have collected fluorescent AuNCs prepared by different ligands, including small molecules, polymers, and biomacromolecules, and highlighted current achievements of AuNCs in biomedical applications for imaging, detection, and therapy. According to these advances, we further provided conclusions of present challenges and future perspectives of AuNCs for fundamental investigations and practical biomedical applications.

## Background

Recent biomedical applications have revealed the significant roles of nanomaterials in the developments of nanoscience and nanotechnology [1–10]. In comparison with bulk materials, nanomaterials have shown unique physical and chemical properties, making them promising building blocks [11–18]. Among the different nanomaterials, a specific type of gold nanomaterials, gold nanoclusters (AuNCs), with sizes up to hundreds of gold atoms have been extensively investigated in biomedical applications due to their well-defined structure, facile surface modification, and highly stable optical property [19–34]. Without a distinct surface plasmon resonance, AuNCs have exhibited fluorescence in the broad region from visible to near-infrared with long lifetime and large Stokes shift [35–37]. Great efforts have been made for the uses of AuNCs as the fluorescent probes in biomedical applications for the fields of imaging, detection, and therapy [38–40]. In comparison with organic fluorophores and quantum dots, fluorescent AuNCs have shown high compatibility, superior photostablility, and excellent water solubility for long-term imaging, high-sensitivity detection, and target-specific treatment [41–49]. The intensive developments of AuNCs as fluorescent probes have brought significant impacts in the applications of imaging, detection, and therapy.

The extensive developments of AuNCs in biomedical applications have been achieved in recent years. Several outstanding review papers of AuNCs with the point of view in analytical applications have been focused on the analyses of drugs, environmental contaminants, and biological samples [50–53]. In this review, we emphasized on the recent advances for the uses of AuNCs conjugated with three types of ligands including small molecules, polymers, and biomacromolecules in the applications for imaging, detection, and therapy. Relevant challenges and future perspectives of AuNCs for fundamental researches and biomedical applications were also provided in the “Conclusions.”

## Small Molecule-Conjugated AuNCs

**Fig. 1:**
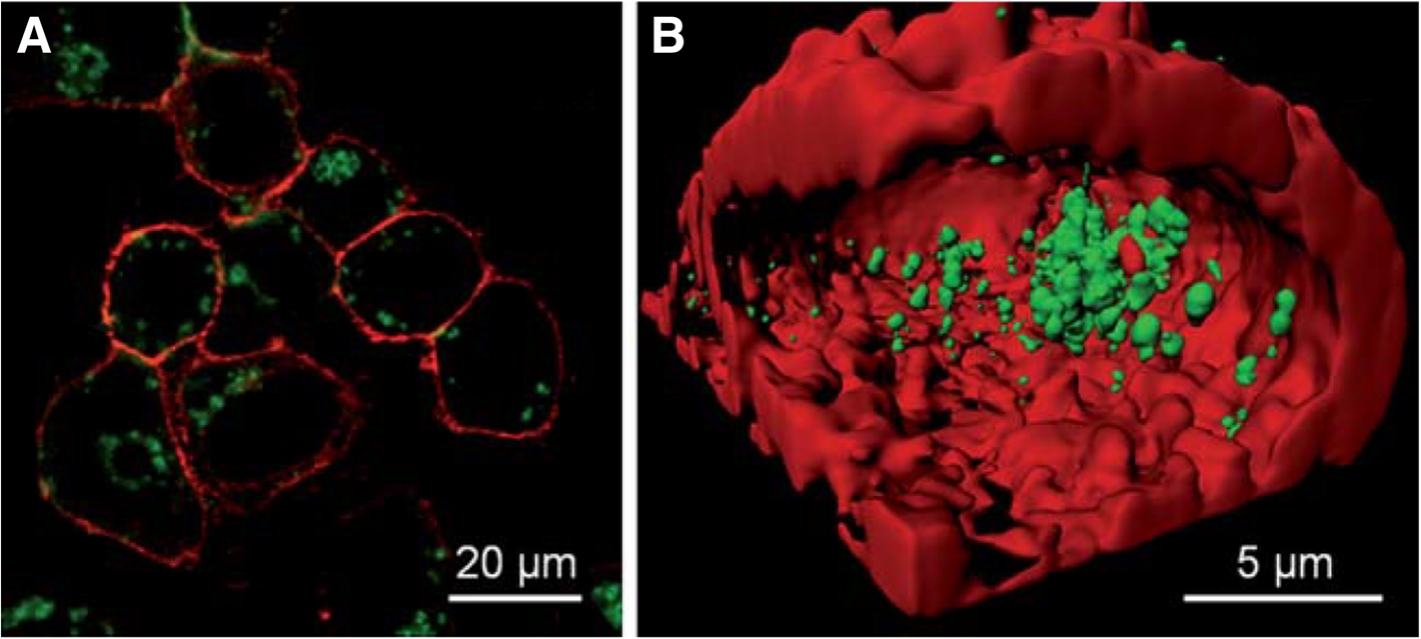
**a** Image of HeLa cells after incubation with DPA-AuNCs for 2 h by confocal microscopy. **b** 3D Image displaying internalized DPA-AuNCs in cross-sectioned view [55]. The colors of DPA-AuNCs and membrane dye DiD are depicted in green and red, respectively

Small molecules have been extensively applied as ligands to prepare AuNCs. With the conjugations of small molecules on the surfaces, AuNCs have exhibited different functions for imaging and detection. For example, d-penicillamine conjugated with gold nanoclusters (DPA-AuNCs) possesses pretty good characteristics such as small size, high colloidal stability, and brightness which imparts them with an immense perspective as fluorescent probes and thus can be utilized for biological imaging. Human cancer (HeLa) cells were imaged by internalization of DPA-AuNCs. Then, after 2 h of incubation of cancer cells with DPA-AuNCs, confocal microscope was used for imaging the cells with two-photon excitation technique [54]. The membrane dye DiD was used as a reference, and the emission intensities of both DPA-AuNCs and DiD dye were collected in green and red colors, respectively. The bright luminescence emitted by the HeLa cells due to ingestion of nanoparticles is shown in Fig. 1a. Also, for 3D rebuilding, different images at various z-positions were taken as shown in Fig. 1b [55].

**Fig. 2:**
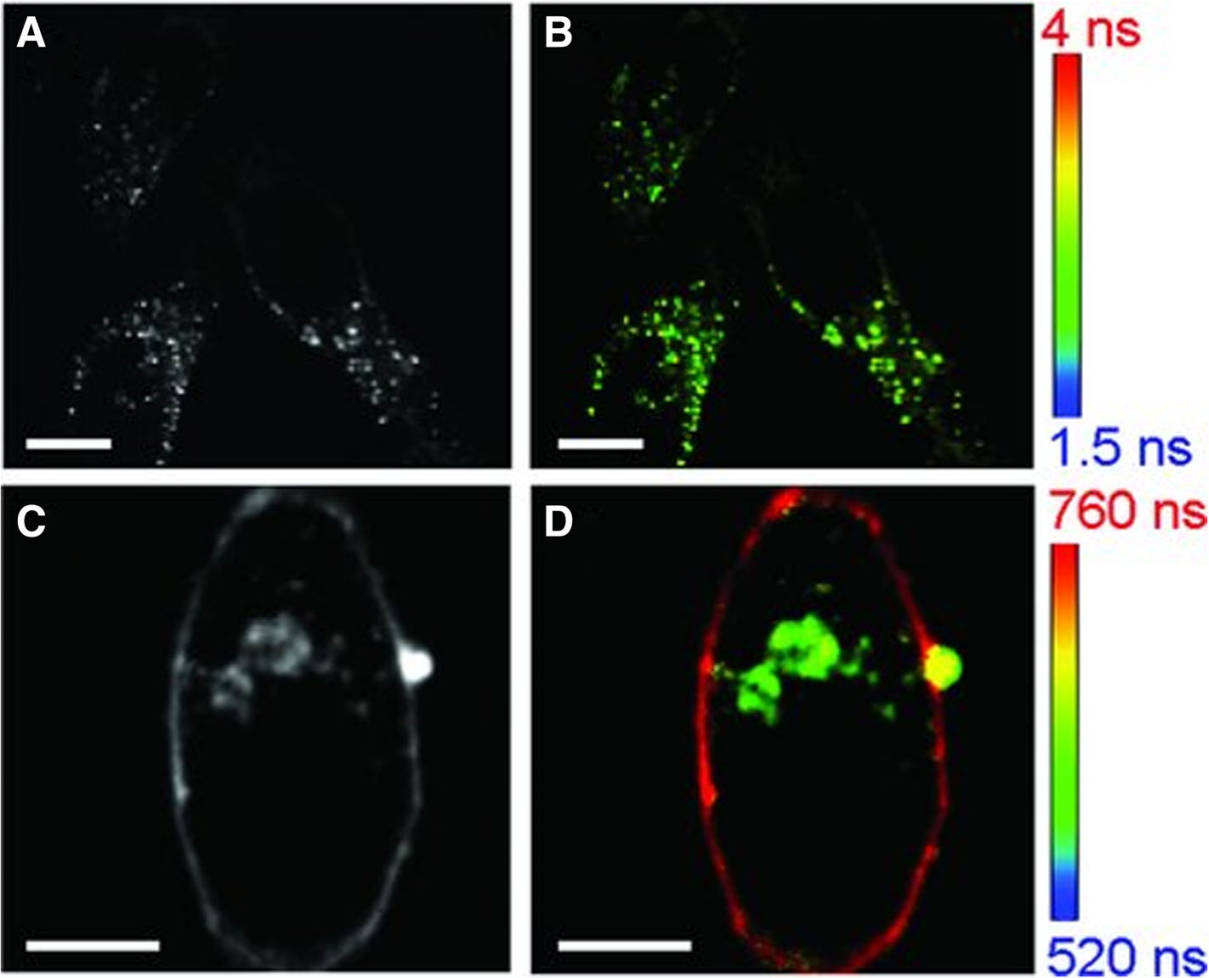
Intensity (**a**, **c**) and FLIM (**b**, **d**) images of Hela cells only (**a**, **b**) and Hela cells incubated with DHLA-AuNCs for 1 h (**c**, **d**). All scale bars are 10 μm [56]

The dihydrolipoic acid (DHLA)-AuNCs were internalized in HeLa cells for investigating the fluorescence lifetime imaging (FLIM) application for the first time. Hela cells without DHLA-AuNCs showed auto-fluorescence with a lifespan between 1.5 to 4 ns. The intensity and lifetime images of Hela cells are shown in Fig. 2a, b. But after exposing Hela cells to DHLA-AuNCs for 1 h, the cells were marked luminescent emittance which exhibited a long fluorescence lifetime of 500 to 800 ns. The intensity and FLIM images of Hela cells with DHLA-AuNCs are shown in Fig. 2c, d [56].

**Fig. 3:**
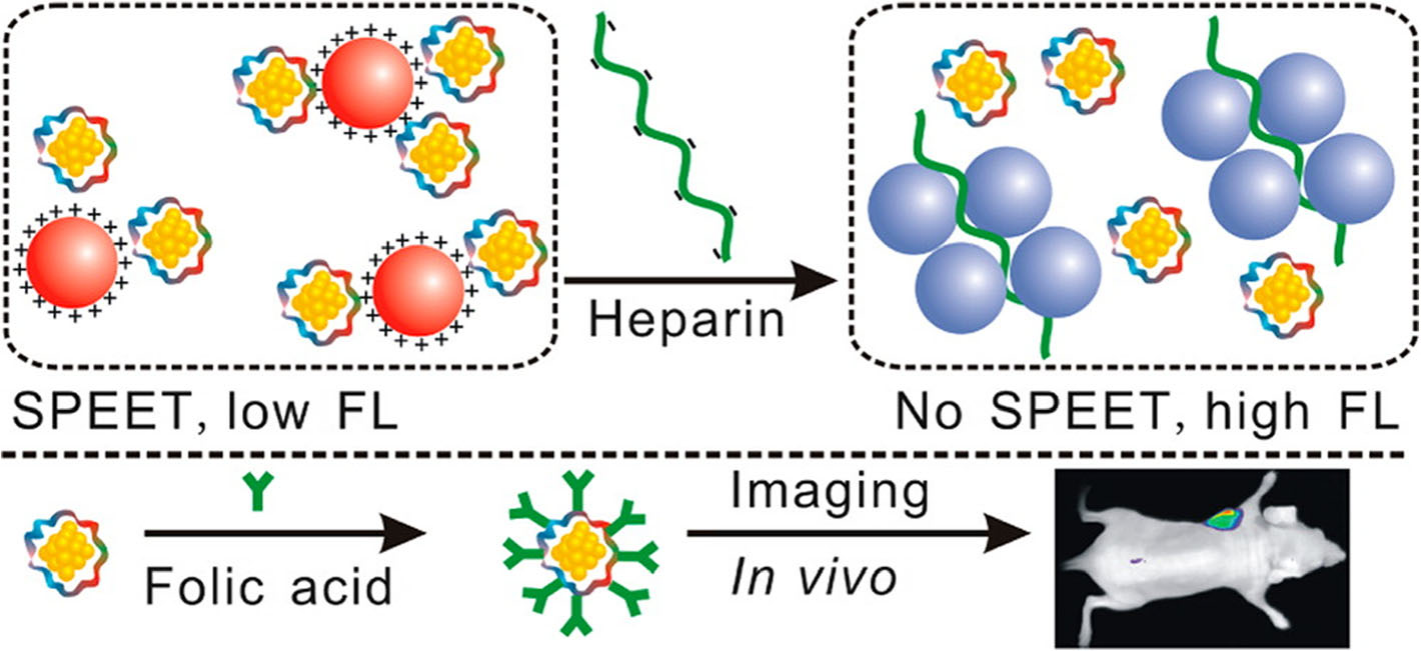
Near-infrared fluorescent try-AuNCs as surface plasmon-enhanced energy transfer biosensor and in vivo cancer imaging bioprobe [58]

Wang et al. found that when cancerous cell lines such as HepG2 (human hepatocarcinoma cell line), K562 (leukemia cell line) was incubated with chloroauric acid (a biocompatible molecular Au(III) species) solutions in micromolar concentrations, and AuNCs get spontaneously biosynthesized by these cell lines [57]. But the phenomenon did not happen in non-cancerous cell line, L02 (human embryo liver cells) which were utilized as controls. As a result, the abovementioned method can be subjugated as a novel method for in vivo self-bio-imaging of tumors. Another trypsin-stabilized gold nanoclusters (try-AuNCs) possessing near-infrared fluorescence were synthesized by Liu et al. for dual purposes; one of the applicability includes biosensing of heparin which is built on surface plasmon-enhanced energy transfer (SPEET) and another includes folic acid (FA)-modified try-AuNCs for in vivo cancer fluorescence imaging (Fig. 3). The SPEET mode and in vivo cancer imaging with high targeting ability possessed by try-AuNCs showed an immense potential as multifunctional biomaterials for biosensing biomolecules [58].

The o-quinone-containing ligands are known to form complexes with Ferric (Fe^3+^) ions [59, 60]. So the AuNCs containing dopaquinone as ligands were developed and evaluated by Ho et al. for the sensing of Fe^3+^ based on a mechanism of the formation of a complex between Fe^3+^ ions and o-quinone moiety of dopaquinone in solution. It was found from the studies that a large complex is formed with dimensions more than 500 nm through aggregation of AuNCs in the presence of Fe^3+^ ions. Thus, AuNCs can be used for detection of Fe^3+^ ions in water and other liquids [61].

**Fig.4:**
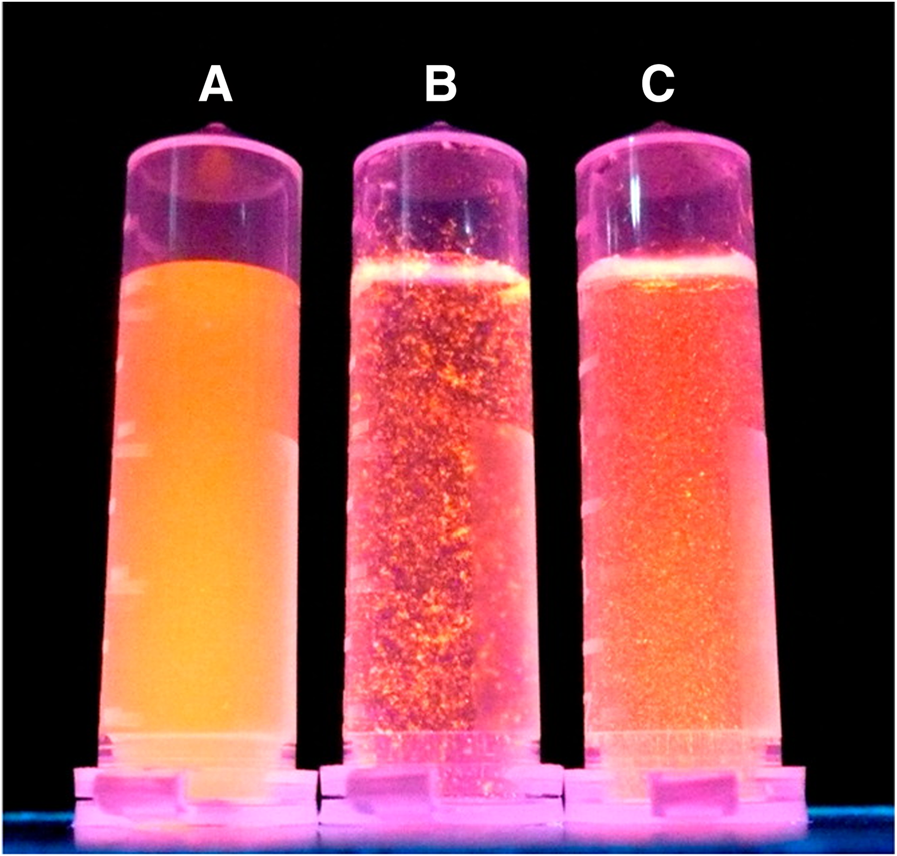
**a** Fluorescence intensity of MUA-AuNCs in the absence of 170 μM Hg^2+^. **b** Agglomeration of Hg^2+^ with COOH group of MUA-AuNCs in the presence of 170 μM Hg^2+^. **c** Fluorescence intensity after 10 mM cysteine had been added to the sample in B [64]

Acidic functional groups are reported to form a stable complex with metal ions and biothiols; similarly, 11-mercaptoundecanoic acid-conjugated gold nanoclusters (MUA-AuNCs) were thought to sense Hg^2+^ ions in solutions and bithiols which can be regarded as one of the sensing applicability of AuNCs [62, 63]. The fluorescence intensity of MUA-AuNCs in complex with Hg^2+^ ions is shown in Fig. 4 [64]. Furthermore, the complex of Hg^2+^-thiol was reported to be more stable than Hg^2+^-COOH complex [65]. Therefore, complex of MUA-AuNCs was used to detect bithiols which can be further regarded as it is another application in sensing of metal ions in various solutions [64].

The vancomycin stabilized gold nanoclusters (Van-AuNCs) were designed and synthesized by Yu et al. for detection of Fe^3+^ in tap water, lake water, river water, and sea water as one of its application in environmental sample analysis [66]. Chitosan-functionalized gold nanoclusters (AuNCs@Chi) were produced and developed for the use as a detecting material of hydrogen sulfide (H_2_S) employing Förster resonance energy transfer (FRET) mechanism [67]. The reason for the researchers to detect H_2_S is that hydrogen sulphide is involved in many biological processes which include vasodilation [68, 69], anti-inflammation [70, 71], and neurotransmission [72].

Liu et al. laid down a foundation for the synthesis of glutathione (GSH)-stabilized gold nanoclusters (GSH-AuNCs) having high selectivity, rapid response, and excellent photostability which were utilized for the detection and sensing of lysine and cysteine (amino acids) [73]. In recent times, FRET (Forster resonance energy transfer) assembly has been developed by Yu et al. using gold nanoclusters capped by glutathione (AuNCs@GSH). The assembly was found to be highly selective for amino acid cysteine which may be employed in future for diagnosing of cysteine-related diseases [74]. The cysteine-rich protein-templated AuNCs were prepared using silver(I) ion. Keratin is a cysteine-rich structural proteins found abundant in hair, wool, feathers, etc. Therefore, silver ions based keratin-templated AuNCs were synthesized and evaluated for their sensing application of mercury ion (Hg^2+^) [75]. Based on dual-emission carbon dots-gold nanoclusters (C-AuNCs) functionalized with dithiothreitol (DTT), a ratiometric fluorescence sensor for the sensitive detection of mercury ions (Hg^2+)^ in water samples has recently been reported [76]. The above two reported applications of AuNCs may act as of great significance for monitoring the quality of water. Cyclodextrin-capped AuNCs have been reported for detection of cobalt ions (Co^2+^) and display fluorescence-based selective and sensitive Co^2+^ ion sensing. Cellular internalization of AuNCs was also observed during the sensing of Co^2+^ ions [77].

Recently, the ultrasmall AuNCs conjugated with biocompatible surface ligand of GSH have been synthesized as the metabolizable and efficient radiosensitizers for cancer radiotherapy [78]. The ultrasmall nanoconstructs of GSH-AuNCs have revealed attractive properties included strong radiotherapy enhancement from the Au core and good biocompatibility from the surface coating GSH. Moreover, the GSH-AuNCs have been preferentially accumulated in tumor via the improved enhanced permeability and retention effect leading to strong enhancement for cancer radiotherapy than that of much larger gold nanoparticles. The enhanced radiotherapy can be attributed to the fact that the DNA damage caused by the photoelectric effect and Compton scattering of the Au_25_ nanoclusters. The remarkable decrease in the volume and weight of U14 tumor has been achieved by using the GSH-AuNCs as the radiosensitizer. Furthermore, after the treatment, the GSH-AuNCs can be efficiently cleared by the kidney, minimizing any potential side effects because of the accumulation of Au_25_ nanoclusters in the animal models.

## Polymer-Conjugated AuNCs

**Fig. 5:**
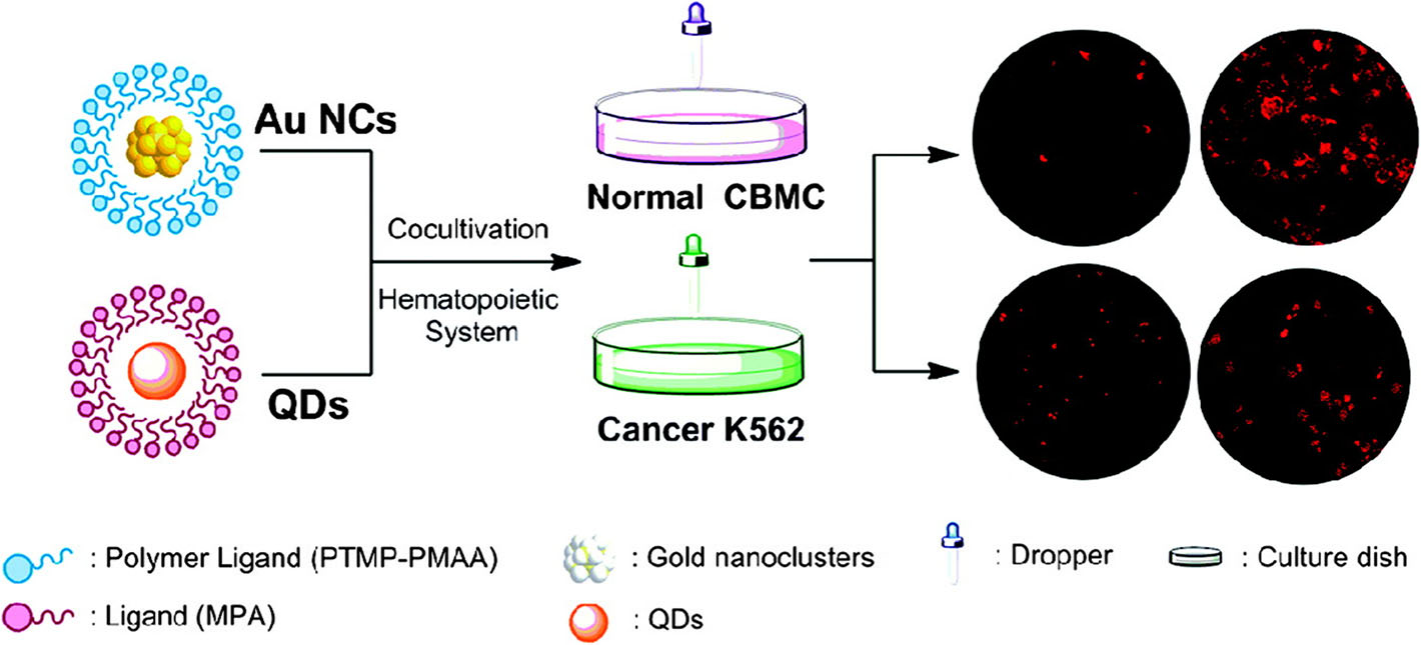
Labelling of normal and cancer cells with AuNCs and quantum dots (QDs) [79]

Polymers have also emerged as the important ligands for preparations of AuNCs in biomedical applications. For example, AuNCs were prepared by capping with multidentate thioether-terminated poly(methacrylic acid) (PTMP-PMAA) ligand which were found to be highly photostable candidates and were used to label the normal (cord blood mononuclear cells; CBMC) and hematopoietic cells (K562 cancer cells) (Fig. 5) [79]. It was revealed from the results that cancer cells ingested these molecules to a much larger extent than normal cells [80]. It has been reported [81] that gold nanoparticles are easily penetrable to more mature cells such as granulocytes and lymphocytes which are part of hematopoietic system. Similarly, AuNCs can also be applicable in selective labeling, imaging, and target drug delivery in the hematopoietic system and related cancers such as chronic myeloid leukemia.

**Fig. 6:**
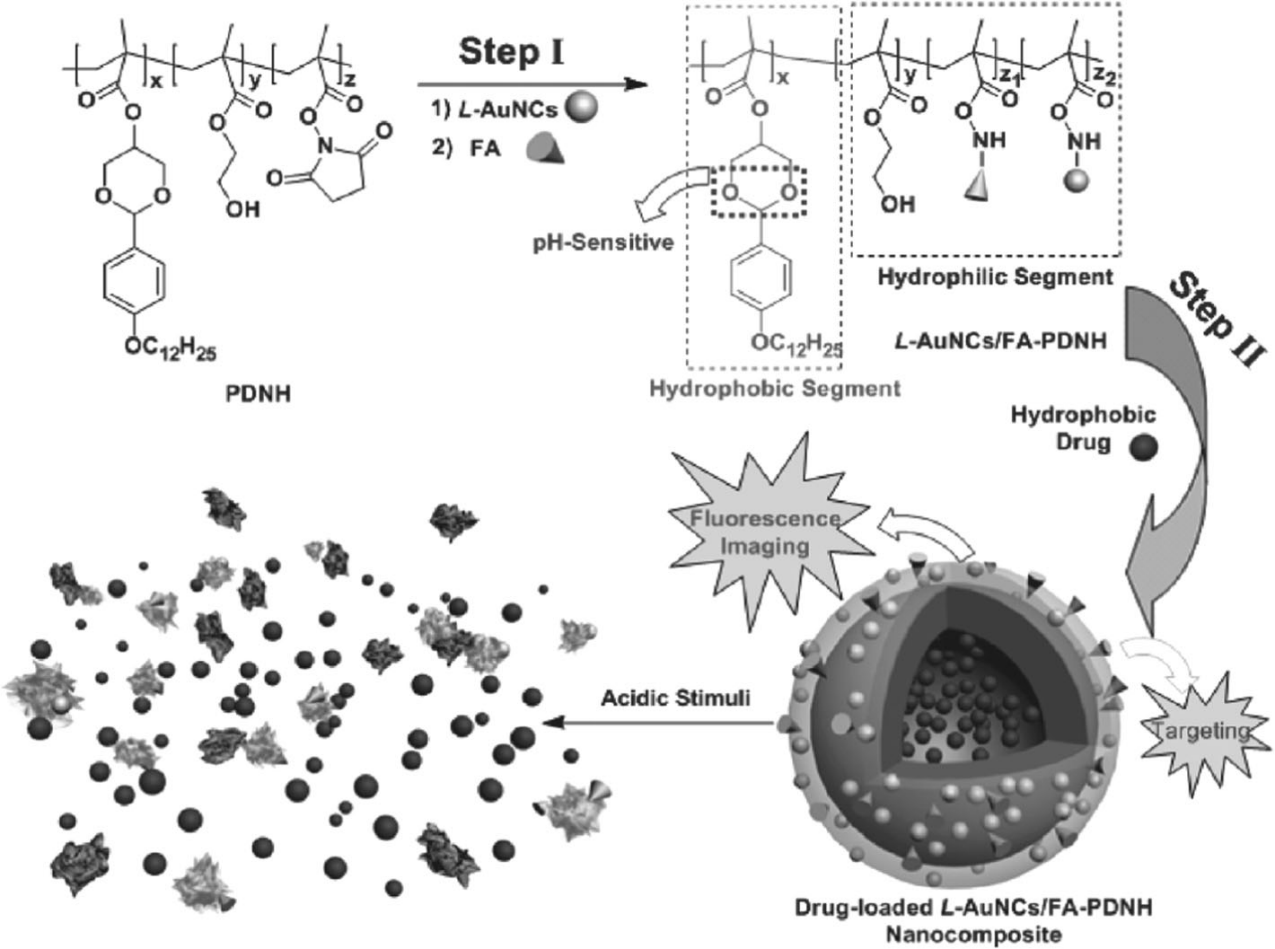
Fabrication of L-AuNCs/FA-PDNH nanocomposite for imaging and therapy [88]

Aldeek et al. designed fluorescent polyethylene glycol- and zwitterion-functionalized gold nanoclusters by using bidentate ligands made of lipoic acid anchoring groups conjoined with either a poly(ethylene glycol) short chain or a zwitterion group [82]. To determine the role of these nanoclusters in biology, various tests were performed like pH-dependent stability and stability in the presence of excess salt. The hypothesis given by the author reveals that these tests are in relevancy to the use of these AuNCs as fluorescent platforms for imaging and sensing in biology. It is further described in the report that various biological abnormalities are related to pH and thus can provide an indication for progression of several diseases such as cancer metastasis, chronic fatigue, and depression [83, 84]. These clusters are also thought to manage the physical behavior of proteins and nucleic acids [85–87]. One of the additional benefit of these clusters is their use in in vivo (deep-tissue) imaging. Chen et al. developed a pH-dependent amphiphilic polymeric system containing luminescent AuNCs that were found to be photostable and biocompatible in the form of nanocomposite for diagnostic activities which includes detection and therapy of folate over-expressing cancerous cells [88]. Luminescent AuNCs were restrained with amphiphilic copolymer (poly(DBAM-co-NASco-HEMA) (PDNH)) to form L-nAuNCs/FA-modified PDNH (or L-AuNCs/FA-PDNH) nanocomposite. Furthermore, hydrophobic drug paclitaxel was assembled with L-AuNCs/FA-PDNH and thus can be utilized for both imaging and treatment of cancer (Fig. 6).

**Fig. 7:**
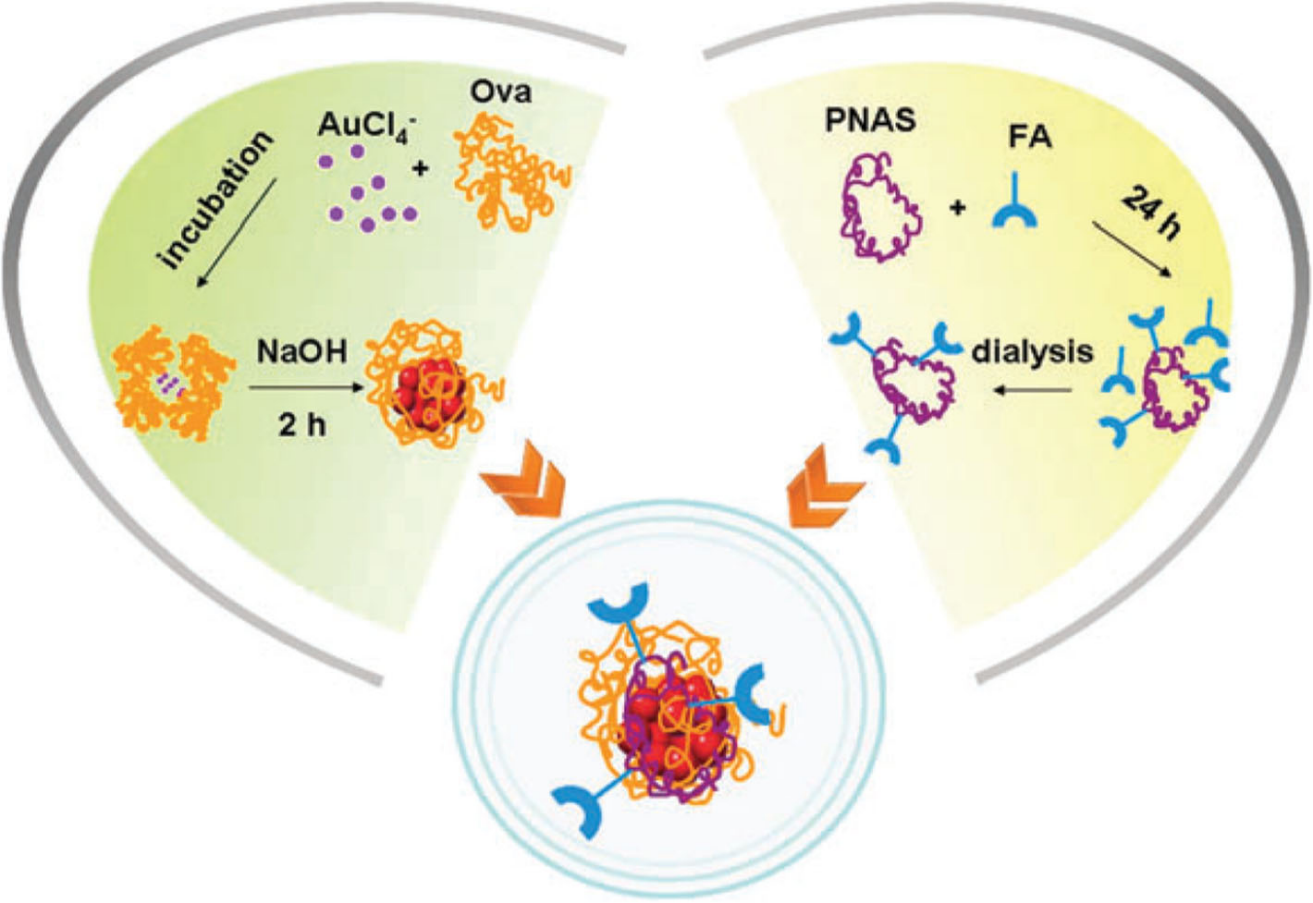
Schematic of the formation of FA-Ova-AuNCs for cancer cell imaging [90]

Polycations-functionalized water-soluble polyethyleneimine gold nanoclusters (PEI-AuNCs) were designed and synthesized for appropriate and safe gene therapy applications along with cell imaging [89]. Due to the enthralling optical properties of PEI-AuNCs, these clusters are reported as a promising candidate for bioimaging, which was confirmed by incubating cancer cell lines (HepG2) with PEI-AuNCs and showed remarkable photoluminescence and the cells giving strong intense red fuorescence. Gold nanoclusters protected by ovalbumin (fluorescent probe) linked with folic acid (targeting ligand) (FA-Ova-AuNCs) and a homopolymer *N*-acryloxysuccinimide as the linker is being developed by Qiao et al. and was utilized for the detection of cancer through cancer cell imaging (Fig. 7). As folic acid receptors are over-expressed in HeLa cells, it is thought that Hela cells would ingest FA-Ova-AuNCs. In this work, specific staining of HeLa cells by FA-Ova-AuNCs has been demonstrated [90].

For the application in detection, highly phosphorescent molecular gold(I) cluster in a macroporous polymer film has been designed and synthesized for the detection of cyanide through colorimetric detection technique. The gold nanoclusters can be used to detect cyanide ions in red wine, coffee, juice, and soil. As cyanide is extremely toxic and hazardous and may lead to death [91], there was a need to find highly selective, sensitive, and cost-effective sensors that can help determine the cyanide levels in the environment, water, and food [92]. So, this gold nanocluster may act as benediction which may help to save a number of lives [93].

## Biomacromolecule-Conjugated AuNCs

**Fig. 8:**
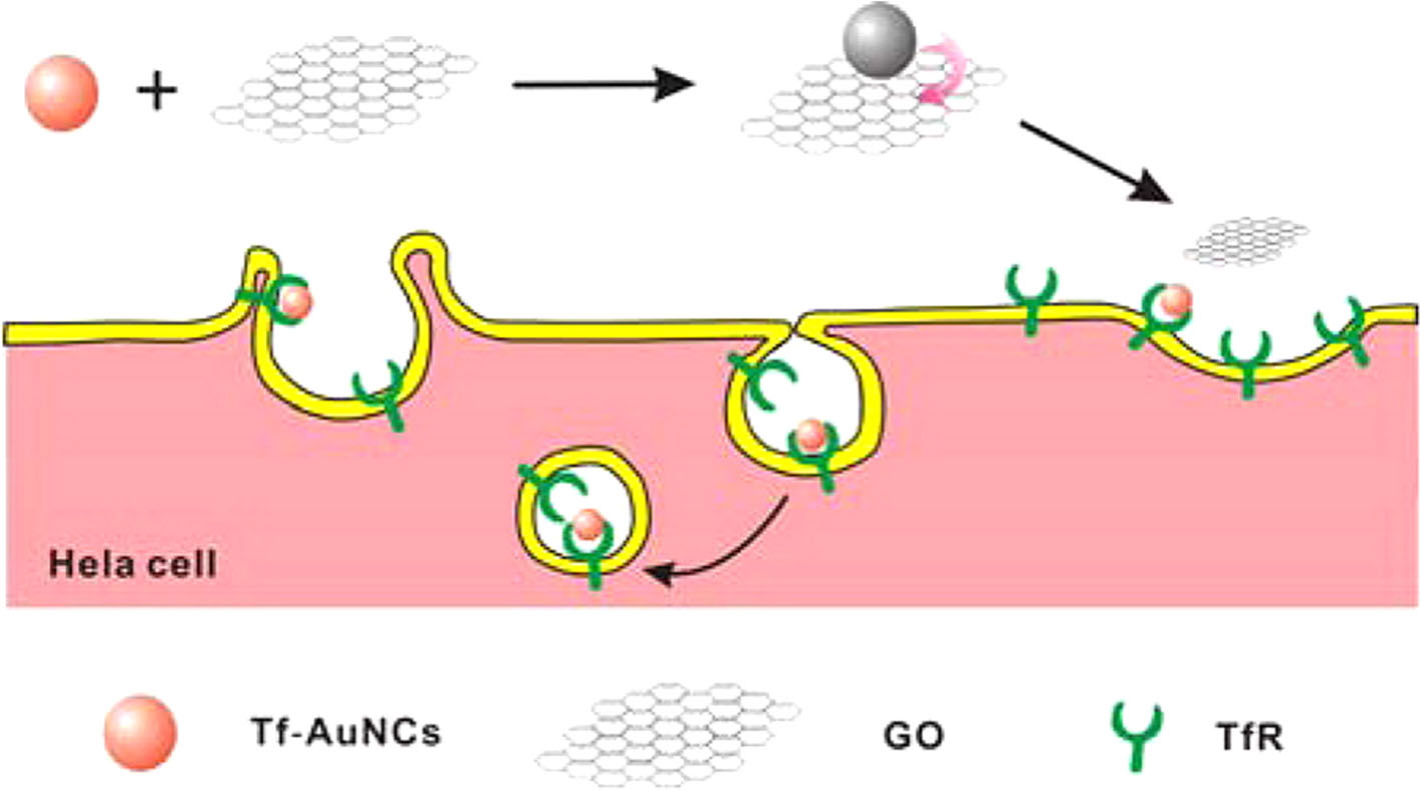
Fabrication of the Tf-AuNCs/GO conjugation as a turn-on NIR fluorescent probe for bioimaging in cancer cells with TfR over expression [94]

Biomacromolecules with thiol groups have also been applied as commonly used ligands to prepare AuNCs in different biomedical applications. Recently, transferrin (Tf)-functionalized gold nanoclusters (Tf-AuNCs)/graphene oxide (GO) nanocomposite (Tf-AuNCs/GO) was manufactured as a turn-on near-infrared (NIR) fluorescent probe which can be utilized for bioimaging of cancer cells and small animals [94]. The ability of NIR fluorescent probe for imaging Tf receptor (TfR) on cancer cells was evaluated using two different cancer cell lines, i.e., Hela (high expression level of TfR) and HepG-2 (low expression level) and one normal mouse cell line (3T3) with different levels of TfR as shown in Fig. 8. The fluorescent probe was obviously ingested only by Hela cells, and noticeable fluorescence was observed after 4 h of incubation.

**Fig. 9:**
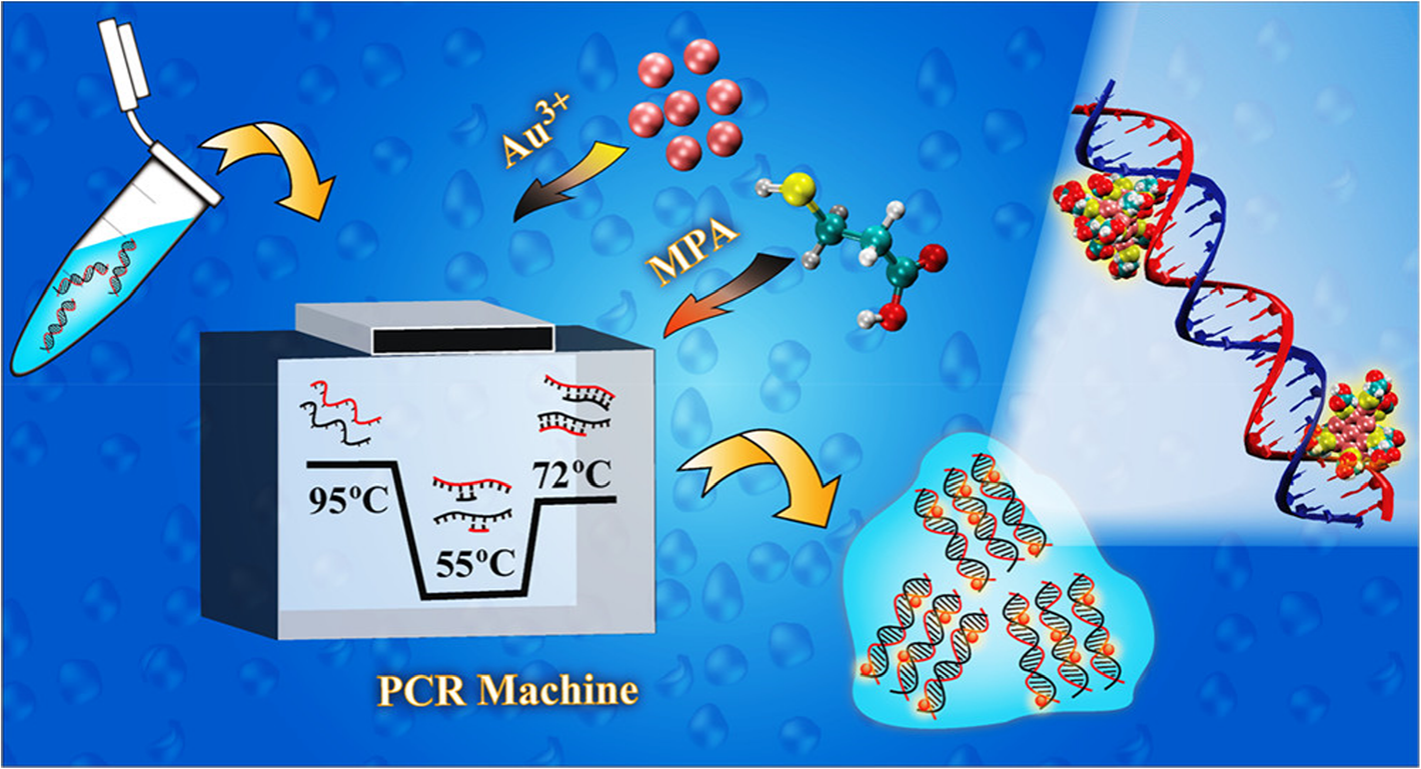
The method for the synthesis of luminescent AuNCs by emulating the PCR condition [95]

Sahoo et al. have developed a quick one-step green synthesis (2 min) of highly luminous AuNCs on DNA, using a single heating and cooling cycle as in polymerase chain reaction (PCR). The intensity of luminescence nanoclusters was found to increase with the number of DNA, offering an easy way to quantify DNA (Fig. 9). As a powerful fluorescent probe for DNA quantification, the capability of AuNCs is shown in two different cancer cell lines included HeLa and A549 [95]. The formation of AuNCs was found to be influenced by the amount of precursors (HAuCl_4_) used in synthesis. The intensity of luminescence emissions and the quantum results of nanoclusters are seen based on the cluster dimensions formed on various amounts of gold. The AuNCs were prepared by different base pairs, which consisted of A, T, G, and C, and produced the same luminescence for different base pair compositions and the same sequence lengths. Furthermore, the identification of the emission-intensity dependence of nanoclusters on DNA quantities provides a unique way of testing. Analysis of gene amplification and relative expression can be obtained. Moreover, the biocompatibility of AuNCs further emphasizes its use as a probe compared with the traditional cytotoxic properties of dyes. Quantitative analysis of the level of gene expression in various cancer cell lines can be used to demonstrate a simple, portable, and low cost equipment as an alternative to complicated, powerful, and expensive PCR energy machine. Furthermore, with the uses of luminescent AuNCs as signal-generating agents, this tool enables reverse transcriptase PCR and array-based analysis of multiple genes/proteins at the same time using switchable holders and specially designed software. Devices and approaches were developed to assess gene profiles associated with apoptosis in HeLa cancer cells and also to measure the expression of glutathione-*S*-transferase (GST) protein and GST-tagged human granulocyte macrophage colony-stimulating factor (GSThGMCSF) recombinant protein extracted form *Escherichia coli* [96].

High-speed preparation of biocompatible signal-producing agents of AuNCs in DNA and proteins allows qualitative detection. Moreover, it provides synthetic methods of AuNCs as common probes for both DNA and protein studies (in fluids as well as samples on membranes), PCR amplicon analysis, and membrane-based researches in a single instrument. The instrument is capable of delivering 95% PCR amplification efficiency as compared to commercially available machines. Most important, the materials are all environmentally friendly. Taking the advantages, integrated tool and approach can create a novel application to existing techniques with the incorporation of nanotechnology and biology.

Nguyen et al. develop double ligand of stabilizing AuNCs and fabricate AuNCs/graphene nanocomplex as a “turn-on” fluorescent probe to detect matrix-related matrix metalloproteinase-9 cancer [97]. A smooth, one-step method was investigated for the biomedical application of AuNCs using peptides and mercapto undecanoic acid as co-templating ligands. The peptide with metalloproteinase-9 cleavage site serves as a stabilizer and also as a targeting ligand for enzyme sensing. With enzymes, because of the excellent quenching properties and negligible background of graphene oxide, the AuNCs/graphene nanomaterial produces a strong “turn-on” fluorescent response, which is highly correlated with enzyme concentrations. The limit of detection of the nanomaterial is 0.15 nM for enzyme. The fluorescent nanomaterial was successfully demonstrated for detection of “turn-on” metalloproteinase-9 secreted from MCF-7 cancer cell with high sensitivity and selectivity. Furthermore, the fluorescent AuNCs provide significant reductions in time, cost, and sensory complexity compared to previous studies. The platform has also shown great potential for detecting different biological molecules in diverse fields including environmental and analytical researches. Similarly, Song et al. develop the label-free, sensitive, and simple method for detecting protein kinases based on the selective aggregation of phosphorylated-gold nanoclusters peptides (AuNCs-peptides) induced by the coordination of Zr ion [98]. The AuNCs were prepared by peptides without a strong reducing agent, which prevents peptides from being disturbed. A study of label-free, green, sensitive, and simple fluorescence using the AuNC-peptides to measure the activity of the protein kinase CK2 has been developed. Compared with the recent established kinase fluorescence test, the uses of AuNC-peptides have several important advantages, including label-free, green, and simple experimental processes.

Selvaprakash et al. develop AuNCs using low-cost chicken egg white proteins (AuNCs@ew) as a switch-on sensing probe to detect phosphate-containing metabolites such as adenosine-50-triphosphate (ATP) and pyrophosphate (PPi) [99]. A cost-effective and straight-forward approach to producing fluorescent AuNC probes for phosphate-containing molecules such as ATP and PPi has been obtained. By adding cheap egg whites with tetrachloroauric, AuNCs@ew can be easily synthesized by microwave heating. In this work, AuNCs@ew mainly dominated by AuNCs@ovalbumin through careful characterization. Since ovalbumin is a glycoprotein and contains abundant glycine ligands, the possibility for the use of AuNCs@ew as the fluorescent probes for ConA, which contains the glycans binding site, has been successfully proven in Selvaprakash’s work.

Wu et al. use bovine serum albumin (BSA) and GSH to synthesize gold nanoclusters (BSA/GSH-AuNCs) with excitation and emissions at 330 nm and 650 nm, respectively [100]. In this approach, BSA and GSH serve primarily as a limitation and reducing agents, respectively. With the help of GSH, only 30 μM BSA is needed to synthesize photostable BSA/GSH-AuNCs. With the use of GSH, the use of large amounts of expensive proteins such as BSA and transferrin is no longer necessary for the development of fluorescent proteins/GSH-AuNCs. This strategy provides a low-cost approach for the synthesis of protein-AuNCs and also simplifies the refining of the established AuNCs. Wu et al. also found that quenching triggered by NO_2_^−^ at pH 3.0 was efficient and specific. With high salt tolerant, sensitivity, and selectivity, BSA/GSH-AuNCs have great potential for measurement of complicated NO_2_ samples. Cao et al. investigate pH-induced fluorescence changes from AuNCs@BSA and appropriate conformational changes of ligand proteins by fluorescence, circular dichroism (CD), and IR spectral measurements. In this work, BSA in AuNCs@BSA undergoes identifiable conformational changes at the level of secondary and tertiary structures. CD and IR results interpret a significant change from the second structure on extreme acidity and alkaline, where more irregular structures are obtained [101]. The difference in secondary structural change trends between AuNCs@BSA and the original BSA was shown. The extreme alkaline condition (pH 11.43) induces a change from exposure to the buried helix. Moreover, the large tryptophan fluorescence gap between AuNCs@BSA and the original BSA implies that the gold nuclei live near tryptophan in the BSA. This study lays the groundwork for understanding the behavioral conformation of ligand proteins in conjugated AuNCs.

Ghosh et al. investigate the effects of AuNCs on CD and enzymatic activity of α-chymotrypsin (ChT) (against substrate hydrolysis, *N*-succinyl-l-phenylalanine p-nitroanilide) [102]. The CD spectrum shows that on binding to AuNCs, ChT is completely exposed, producing almost zero ellipticity. The ChT-coated AuNCs show virtually no enzymatic activity. The additional GSH or oxidized GSH restores enzyme activity a ChT by 30–45%. The activity of ChT is irreversibly lost on the binding surface of AuNCs. This lost activity can be recovered when ChT closes AuNCs treated with GSH or oxidized GSH. In the cell, the enzyme activity can be revived by GSH as shown in this work. Since cancer cells are characterized by elevated levels of glutathione, there will be differences in the absorption of enzyme-lined gold groups between cancer cells and normal cells.

The novel technique of fluorescence-guided surgery has entered the surgical process to help operators make the decision whether the tissues need to be resected or preserved during surgery [103]. These achievements could establish a paradigm shift in cancer surgery for great improvement of patient outcome. Recent research progress in this field has focused on the use of fluorescent AuNCs conjugated with diatrizoic acid and target-specific AS1411 aptamer as a fluorescence-guided probe for providing precise guiding during tumor tissue resection. In vivo experiments have demonstrated that the tumor location in CL1-5 tumor-bearing mouse has been observed from the clear CT image using the AuNC conjugates as a molecular imaging contrast agent. More importantly, the clearly visible orange-red fluorescence of AuNC conjugates has been utilized to help the resection of the CL1-5 tumor by intraoperative fluorescence guidance. The strong fluorescence enhancement of resected tumor is based on in vivo imaging system data to prove the successful molecular targeting using fluorescent AuNC conjugates. This work has demonstrated great advantages of using the target-specific fluorescent AuNC conjugates in vivo that are able to provide long-term fluorescent imaging times, high photostability, dual imaging functions, and feasible surface modifications with specific-targeted molecules in comparison to most organic contrast agents currently used. In addition, this work has brought an advanced concept in the field of biomedical imaging and therapeutics using functionalized AuNCs.

## Conclusions

Overall, we have provided a mini review of the recent advances in fluorescent AuNCs prepared with small molecules, polymers, and biomacromolecules for the applications in bioimaging, detection, and therapy (Table 1). These works have shown that fluorescent AuNCs can be promising fluorescent probes due to their unique properties such as excellent biocompatibility, high photostablility, and easy surface modification. Although AuNCs have been demonstrated in various biomedical applications, however, their fluorescence quantum yields (QYs) are still low (usually less than 20%). The first challenge to extend the applications of AuNCs is focused on the preparation of AuNCs with high fluorescence QY. With low fluorescence QY, the synthesis of AuNCs with uniform size will be an alternative way to improve their fluorescence QY. Furthermore, with the uniform size, fluorescent AuNCs with a narrow emission spectrum will increase their benefit in biomedical applications. The second challenge for AuNCs is the control of ligand on their surface because the chemical and physical properties of AuNCs can be significantly affected by their surface modification. Therefore, the theoretical and practical studies of AuNCs are still needed to have a better understanding of their structure, optical characteristic, and physicochemical property. Especially, for physicochemical property, recent studies have proven that AuNCs are potential fluorescent probes for biosensing, bioimaging, and cancer therapy. Accordingly, to realize the biomedical applications, we still have a lot of works to push the biomedical applications of AuNCs in imaging, detection, and therapy. Overall, with the great efforts, we believe that AuNCs will be served as a significant fluorescent probe in biomedical application in the near future.

**Table 1:** Colors, detection targets, and applications of AuNCs with various ligands in this review

Type	Ligand	Color	Target	Application	Ref.
Small molecules	d-Penicillamine	Orange-red	HeLa cell	Imaging	[55]
	Dihydrolipoic acid	Red	HeLa cell	Imaging	[56]
	Chloroauric acid	Green	Chronic myeloid leukemia	Imaging	[57]
	Trypsin	Red	Hela tumor	Imaging	[58]
	Dopaquinone	Green-yellow	Fe^3+^	Detection	[61]
	11-Mercaptoundecanoic acid	Orange	Hg^2+^	Detection	[64]
	Vancomycin	Blue	Fe^3+^	Detection	[66]
	Chitosan	Green	H_2_S	Detection	[67]
	Glutathione	Red	Lysine	Detection	[73]
	Cysteine	Red	Hg^2+^	Detection	[75]
	Dithiothreitol	Orange-red	Hg^2+^	Detection	[76]
	Cyclodextrin	Orange-red	Co^2+^	Detection	[77]
	Glutathione	Red	U14 tumor	Therapy	[78]
Polymers	Poly(methacrylic acid)	Red	K562 cell	Imaging	[79]
	Poly(ethylene glycol)	Red	Deep tissue	Imaging	[82]
	Poly(DNH)	Orange	HeLa tumor	Imaging and therapy	[88]
	Poly(ethyleneimine)	Pink	HepG2 cell	Imaging and therapy	[89]
	Poly(*N*-acryloxysuccinimide)	Red	HeLa cell	Imaging	[90]
	Poly(acrylonitrile)	Red	CN^−^	Detection	[93]
Biomacromolecules	Transferrin	Red	Transferrin receptor	Imaging	[94]
	β-Actin	Orange-red	DNA	Detection	[95]
	Bovine serum albumin	Orange-red	HeLa cell and *E. coli*	Detection	[96]
	Peptide (GPLGMWSRGLC)	Red	Metalloproteinase-9	Detection	[97]
	Peptide (CCYRRRADDSD)	Violet	CK2 kinase	Detection	[98]
	Chicken egg white proteins	Orange-red	Concanavalin A	Detection	[99]
	Bovine serum albumin	Red	NO_2_^−^	Detection	[100]
	Bovine serum albumin	Orange-red	Bovine serum albumin	Detection	[101]
	Chymotrypsin	Red	Chymotrypsin	Detection	[102]
	AS1411 aptamer	Orange-red	CL1–5 tumor	Imaging and therapy	[103]

## Abbreviations

AuNCs: Gold nanoclusters
BSA: Bovine serum albumin
CBMC: Cord blood mononuclear cells
CD: Circular dichroism
Chi: Chitosan
ChT: Chymotrypsin
DHLA: Dihydrolipoic acid
DPA: D-penicillamine
DTT: Dithiothreitol
FA: Folic acid
FLIM: Fluorescence lifetime imaging
FRET: Förster resonance energy transfer
GSH: Glutathione
GST: Glutathione-*S*-transferase
MUA: 11-Mercaptoundecanoic acid
Ova: Ovalbumin
PCR: Polymerase chain reaction
PDNH: Poly(DBAM-co-NASco-HEMA)
PEI: Polyethyleneimine
PPi: Pyrophosphate
PTMP-PMAA: Multidentate thioether-terminated poly(methacrylic acid)
SPEET: Surface plasmon-enhanced energy transfer
Tf: Transferrin
Try: Trypsin
VAN: Vancomycin
